# Effect of Moxibustion on *β*-EP and Dyn Levels of Pain-Related Indicators in Patients with Rheumatoid Arthritis

**DOI:** 10.1155/2021/6637554

**Published:** 2021-04-03

**Authors:** Yingni Wang, Siyu Tao, Zeyun Yu, Yun Luo, Yuan Li, Jie Tang, Guanhua Chen, Rouxian Shuai, Xinyue Hu, Ping Wu

**Affiliations:** ^1^Chengdu University of Traditional Chinese Medicine, Sichuan, Chengdu 610075, China; ^2^Hospital of Chengdu University of Traditional Chinese Medicine, Sichuan, Chengdu 610075, China

## Abstract

**Background:**

Rheumatoid arthritis (RA) is a systemic immunodeficiency disease characterized by persistent synovial inflammation, pannus formation, and bone and cartilage destruction, resulting in joint malformations and function decline.

**Objective:**

The purpose of this study is to evaluate the effect of moxibustion on clinical symptoms and levels of pain-related indicators beta-endorphin (*β*-EP) and dynorphin (Dyn) in patients with RA and to explore the potential anti-inflammatory and analgesic mechanisms of moxibustion in RA treatment.

**Methods:**

A total of 64 patients with RA who met the inclusion criteria were randomly and equally classified into the control and treatment groups. The control group received conventional treatment (oral methotrexate, folate, or leflunomide prescribed for a long time). The treatment group was treated with moxibustion at ST36 (Zusanli), BL23 (Shenshu), and Ashi points with respect to the control group. Patients' clinical symptoms and routine inspection indexes (rheumatoid factor [RF], erythrocyte sedimentation rate [ESR], and C-reactive protein [CRP]) were recorded before and after treatment. Serum levels of tumor necrosis factor-*α* (TNF-*α*), interleukin-1*β* (IL-1*β*), *β*-EP, and Dyn were determined by enzyme-linked immunosorbent assay (ELISA). The software SPSS24.0 was used for statistical analysis.

**Results:**

(1) Compared with the pretreatment result, both of the two groups' clinical symptoms and routine inspection indexes (RF, ESR, and CRP) improved (*P* < 0.05), and the improvement of clinical symptoms in the treatment group outperformed that in the control group (*P* < 0.05). (2) TNF-*α* and IL-1*β* levels decreased significantly in the treatment group after treatment (*P* < 0.01), while no significant difference was observed in the control group (*P* > 0.05). (3) *β*-EP and Dyn levels in the treatment group were significantly increased after treatment (*P* < 0.01, *P* < 0.01), but the control group showed no significant difference (*P* > 0.05, *P* > 0.05). It is worth mentioning that the serum TNF-*α*, IL-1*β*, *β*-EP, and Dyn levels between the two groups were significantly different after 8 weeks of treatment (*P* < 0.05). (4) Differences in the serum *β*-EP and Dyn levels in the patients of the treatment group were correlated with TNF-*α* and IL-1*β* levels after treatment, and the correlation was mainly negative (*r* < 0).

**Conclusion:**

Moxibustion can improve joint pain in patients with RA using conventional western medicine. One of the mechanisms may affect the serum *β*-EP and Dyn levels by downregulating the inflammatory factors to play an anti-inflammatory and analgesic role.

## 1. Introduction

Rheumatoid arthritis (RA) is a systemic immunodeficiency disease mainly characterized by aggressive arthritis. Cytokines such as interleukin-1*β* (IL-1*β*), interleukin-6 (IL-6), and tumor necrosis factor-*α* (TNF-*α*) are active in the joints of RA patients. These cytokines play a crucial role in the pathogenesis of RA, causing inflammation, pain, and joint destruction [1]. About 0.5% to 1% of the global population is affected by RA [2], and patients usually show symptoms such as swelling, stiffness, and deformity of multiple joints. Wrist joints, proximal interphalangeal joints, and metacarpophalangeal joints are most commonly involved [3]. Pain caused by an unrelenting inflammatory response can seriously affect people's quality of life.

The opioid family is widely involved in the regulation of nociceptive sensation, which is mainly divided into endogenous opioid peptides and exogenous opioid peptides. Studies have shown that endogenous opioid peptides are extensively involved in stress regulation and play an important regulatory role between the central nervous system and the immune system [4]. Data from animal and human clinical studies showed the key role of opioid receptors in the regulation of pain and inflammation [5]. Inflammation intensifies the expression, transport, and accumulation of peripheral opioid receptors in sensory nerve endings and triggers the migration of opioid peptides that contain immune cells [6]. Immune cells migrate to inflammatory tissues and release endogenous opioid peptides, which bind to peripheral opioid receptors that produce analgesic effects [7, 8]. Beta-endorphin (*β*-EP) and dynorphin (Dyn) are important endogenous opioids that are involved in the body's response to pain. In the early stage of inflammation (within 6 hours), inflammatory cytokines such as noradrenaline, TNF-*α*, and IL-1*β* stimulate the production of *β*-EP, enkephalins (ENK), and Dyn in leukocytes to activate the surrounding *μ*, *δ*, and *κ* opioid receptors to inhibit nociception [9]. Therefore, the release of endogenous opioid peptides can activate the peripheral opioid receptors to participate in the inhibitory mechanism of pain, which will block further transmission of pain signals, delay the progression of RA disease, and improve the quality of life of RA patients.

At present, western medicine treatments are mostly used for RA. Disease-modifying antirheumatic drugs (DMARDs) are the primary drugs. However, their toxicity and side effects are relatively large, and long-term use of these drugs is likely to harm the functioning of the liver, kidneys, and gastrointestinal system. Studies have shown that moxibustion has a significant impact on the pain of RA patients [10] and can exert immune regulation, anti-inflammatory, and analgesic effects by regulating the expression of inflammatory cytokines, proteins, and related signaling pathways. Moreover, it is easily accepted by patients for the lack of side effects [11–13]. In this study, *β*-EP and Dyn were selected to explore the mechanism of moxibustion on RA and provide a more reliable clinical basis for RA treatment.

## 2. Methods

### 2.1. Design and Setting

A total of 64 RA patients, who fulfilled the inclusion criteria, were included in the outpatient and inpatient collection of the Rheumatology Department of Sichuan Hospital of Traditional Chinese Medicine from March 2018 to December 2019. This research conforms to the Hippocratic Declaration and has been approved by the Sichuan Regional Ethics Review Committee of Traditional Chinese Medicine (No. 2015KL-05). Informed consent was obtained from each of the study participants.

All patients were randomly divided into the control group (32 cases) and the treatment group (32 cases). The patients of the control group were given oral methotrexate, folate, or leflunomide at the doctors' recommendation for a long time. The treatment group received moxibustion at ST36 (Zusanli), BL23 (Shenshu), and Ashi points in addition to conventional medicine, twice a week, 4 weeks for a course of treatment, and two consecutive courses of treatment.

### 2.2. Study Subjects

Participants were recruited by advertisement through hospital official accounts and bulletin boards. Baseline assessment was conducted for all participants who met eligibility criteria, and relevant demographic and general medical data were collected.

#### 2.2.1. Inclusion Criteria

Participants in the study were required to meet all of the following conditions:

1. RA was diagnosed in accordance with the 2010 RA diagnostic criteria developed by the American College of Rheumatology (ACR) and the European League Against Rheumatism (EULAR) [14]
2. Age between 25 and 65 years old, visual analogue scale (VAS) ≥ 3, disease activity score in 28 joints (DAS28) > 3.2
3. Conscious and able to cooperate with the study
4. Did not participate in other clinical trials
5. Sign the informed consent to enter the clinical study

#### 2.2.2. Exclusion Criteria

Participants were excluded from the study if they had any of the following conditions:

1. Unconscious and unable to complete the research
2. With severe joint malformation, stage IV function
3. With other immune system diseases, such as Sjogren's syndrome
4. With malignant tumors and serious diseases such as hematopoietic system
5. Pregnant or lactating women and people with cyclothymic
6. Allergic constitution or drug allergy
7. Afraid of moxibustion therapy
8. Do not take medicine and moxibustion as prescribed

### 2.3. Randomization and Blinding

A random number table generated by the statistical software SPSS24.0 was used to randomly assign eligible participants to the treatment group and the control group in 1 : 1 ratio. Random information was sealed in opaque envelopes, and random operations were supervised by an independent investigator. Because of the particularity of moxibustion therapy, it was easy to know whether moxibustion treatment has been carried out; so it is hardly possible to blind the patients and clinicians. Outcome assessors, data collectors, and statisticians were blinded to the treatment allocation to eliminate potential bias.

### 2.4. Interventions

**Figure 1:**
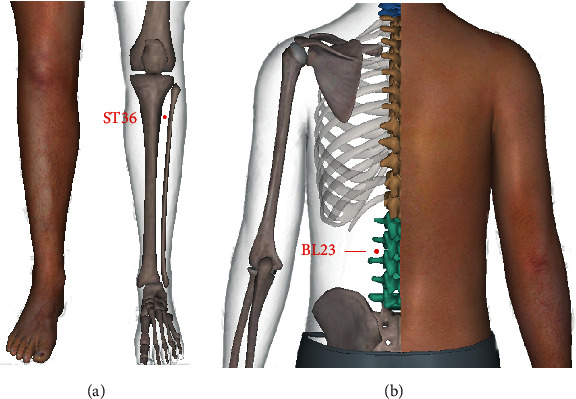
Acupoints. ST36 (Zusanli) is located 3 inches below the outer knee, 1 inch apart from the tibia front margin, BL23 (Shenshu) is located below the second lumbar spine process, 1.5 inches away from the posterior midline, and “Ashi” points are located at where swelling and paining occur.

All patients received oral administration of methotrexate (7.5 mg/dose, 1 dose/week), folate (10 mg/dose, 1 dose/week), or leflunomide (10 mg/dose, 1 dose/day) for a long time. The treatment group was additionally treated by moxibustion at ST36 (Zusanli), BL23 (Shenshu), and Ashi points (Figure 1), which were selected according to the National Standard of the People's Republic of China (GB/T12346-2006). ST36 (Zusanli) and small joints of limbs were applied with moxibustion with seed-sized moxa cone, BL23 (Shenshu) on salt, and large joints on ginger, twice a week, 4 weeks for a course of treatment, and two consecutive courses of treatment. Moxibustion was performed by licensed-TCM doctors with over 3 years of experience in clinical practice.

**Figure 2:**
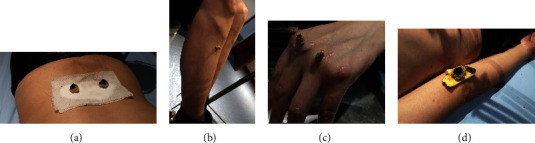
The operation diagram of moxibustion. (a) Participant was treated at BL23 (Shenshu) acupoint. (b) Participant was treated at ST36 (Zusanli) acupoint. (c) and (d) participants were treated at Ashi acupoints.

Doctors marked the acupoints with a marker and then made a moxa cone as big as half olive (about 1 cm in diameter and height), put it on a gauze strip filled with a proper amount of salt, and placed it on both sides of BL23 (Shenshu) points (Figure 2(a)). Direct moxibustion was used for the acupoints of ST36 (Zusanli) and the Ashi points of small joints of the limbs. Doctors applied Vaseline on the corresponding acupoints, put the moxa cone (about 0.3 cm in diameter and height) made with mugwort floss on acupoints, and then ignited the top of it (Figures 2(b) and 2(c)). If patients felt burning pain during the treatment, the cone would be lifted quickly and replaced. The Ashi points of the large joints of limbs were applied with moxibustion with ginger (Figure 2(d)), and the moxa cone was burned on the cut ginger and placed on Ashi points. When the patients felt hot, another ginger would be padded under it until the moxa cone was burned out.

### 2.5. Outcome Measures

Visual analogue score (VAS: measure pain intensity), morning stiffness score, and disease activity score in 28 joints (DAS28: determine disease activity) were used to evaluate the clinical symptoms of the patients before and after treatment. The changes of routine inspection indexes: RF, ESR, and CRP, were compared. The contents of *β*-EP, Dyn, TNF-*α*, and IL-1*β* in serum samples of the two groups were determined by enzyme-linked immunosorbent assay (ELISA). The safety of moxibustion was assessed by the occurrence of adverse events, such as burning, scalding, and blisters.

### 2.6. Specimen Collection

About 3–5 ml of elbow venous blood was extracted from the patients before and after treatment, and the serum was isolated and stored at −80°C. After the course of treatment was completed, the serum samples were sent to Chengdu Lilai Biomedical Experimental Center for testing. The ELISA was used to determine the *β*-EP, Dyn, TNF-*α*, and IL-1*β* levels in these samples. The methods strictly complied with the kit instructions.

### 2.7. Statistical Analysis

The software SPSS24.0 (SPSS, Inc., Chicago, Illinois, USA) was used for statistical analysis. The chi-square (*χ*^2^) test was used for counting data. The *t-*test was used for measurement of data satisfying normal distribution: paired sample *t-*test was used for in-group analysis, and independent sample *t-*test was used for intergroup analysis. Nonparametric test was used for measurement of data that were not normally distributed; Wilcoxon signed-rank sum test was used for in-group analysis, and Mann–Whitney *U* test was used for intergroup analysis. All data were expressed in the form of mean ± standard deviation (Χ ± s). The value of *P* < 0.05 was considered statistically significant, and *P* < 0.01 was considered significantly different.

## 3. Results

### 3.1. Demographics and Baseline Characteristics

**Figure 3:**
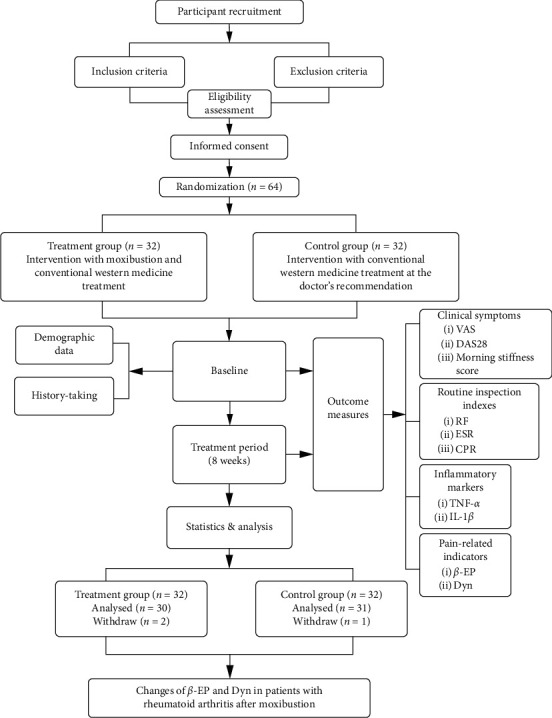
Technology roadmap.

Sixty-four patients were screened and randomly divided into the control group (32 cases) and the treatment group (32 cases). Two participants in the treatment group and one participant in the control group dropped out during the course of the study because of withdrawal of consent (Figure 3). Therefore, a total of 61 patients completed the study. The treatment group consisted of 30 patients. Out of them, 4 were males and 26 were females (with an average age of 53 ± 8.80 years and mean course of disease of 10.11 ± 9.02 years). There were 31 patients in the control group, including 5 males and 26 females (with an average age of 49.39 ± 7.72 years and a mean course of disease of 9.13 ± 9.07 years). There was no significant difference in general data between the two groups (*P* > 0.05).

The baseline characteristics of demographic data, clinical symptoms, and routine inspection indexes of both groups are shown in Table 1.

### 3.2. The Clinical Symptoms and Routine Inspection Indexes

VAS, morning stiffness score, and DAS28 of the two groups were decreased after treatment as compared to that before treatment, and the difference was statistically significant (for VAS, *P* < 0.01 in both groups; for morning stiffness score and DAS28, *P* < 0.01 in the treatment group and *P* < 0.05 in the control group), indicating that clinical symptoms were improved after treatment. The improvement in the treatment group was more significant than that in the control group after treatment, and the difference was statistically significant (*P* < 0.05) (Table 2).

RF, CRP, and ESR were significantly improved after treatment both in the treatment group (*P* < 0.01) and in the control group (RF and CRF, *P* < 0.05; ESR, *P* < 0.01). However, there was no statistically significant difference in RF, CRP, and ESR content between the two groups after treatment (*P* > 0.05), indicating that the routine inspection indexes of the two groups were not significantly improved after treatment (Table 2).

### 3.3. The Contents of TNF-*α* and IL-1*β*

The serum levels of TNF-*α* and IL-1*β* decreased significantly in the treatment group after treatment (*P* < 0.01), while there was no significant difference in the serum levels of TNF-*α* and IL-1*β* in the control group (*P* > 0.05). The levels of TNF-*α* and IL-1*β* in the treatment group were significantly lower than those in the control group, with statistically significant differences between the two groups (*P* < 0.05) (Table 3).

### 3.4. The Contents of *β*-EP and Dyn

The contents of *β*-EP and Dyn in the treatment group were significantly different after treatment (*P* < 0.01, *P* < 0.01), while there were no significant differences in the control group (*P* > 0.05, *P* > 0.05). A significant difference was observed in *β*-EP and Dyn levels of both groups after 8 weeks of treatment (*P* < 0.05) (Table 3).

### 3.5. Correlation Analysis of *β*-EP and Dyn with TNF-*α* and IL-1*β* in the Treatment Group

The differences in serum levels of *β*-EP and Dyn in the treatment group patients were correlated with the differences in TNF-*α* and IL-1*β* after treatment, and the correlation was found to be negative (*r* < 0) (Table 4).

### 3.6. Adverse Events

Only one participant experienced an adverse reaction to skin blisters during the study. The skin condition of the patients improved after the timely treatment of the researchers, and the study was continued.

## 4. Discussion

The aim of the research was to assess the clinical effects of moxibustion on *β*-EP and Dyn levels in the serum of RA patients and reveal the potential mechanism of moxibustion.

### 4.1. The Effect of Moxibustion on Routine Inspection Indexes Related to Clinical Symptoms and Disease Activity in RA Patients

Subjective scales and routine inspection indexes were used in this study to evaluate the activity of the disease so that the therapeutic effect of this study on RA could be more objectively understood. The results of this study showed that pain and other related symptoms of the two groups of patients were improved after treatment, but the improvement was more obvious in moxibustion combined with conventional drug treatment. However, there was no significant difference between the two groups in terms of routine inspection indexes.

Moxibustion has an obvious effect on pain diseases, such as chronic visceral pain hypersensitivity, rheumatoid arthritis, and other conditions. The analgesic effect of moxibustion may be closely related to its thermal effect, infrared radiation effect, regulation of pain-causing factors, inhibition of synaptic mechanism, and central signal integration [15]. ST36 and BL23 are commonly used acupoints in the treatment of RA. Our studies in the early stage also reported that the application of moxibustion on ST36 and BL23 could relieve pain and significantly improve the clinical symptoms of RA patients [11, 12, 16]. A large number of animal experiments have shown that moxibustion has an exact effect on the treatment of ST36 and BL23 points in RA models. It can effectively reduce synovial tissue and fibrous tissue hyperplasia and improve joint swelling and synovial inflammation in the RA models by affecting related proteins, transcription factors, and signaling pathways [13, 17, 18].

This research suggested that moxibustion therapy based on western medicine can give rise to prominent therapeutic effects, improve the clinical symptoms of patients to a greater extent, and reduce the activity of the disease. It may be related to the fact that RA is part of the bi-syndrome of TCM. Moxibustion can warm channels to remove coldness, activate meridians to stop the pain, and have analgesic and anti-inflammatory effects on the pain and swelling of joints in RA patients. The result that no significant difference was found in routine inspection indexes between the two groups can be speculated to be related to the complexity of the disease. As RA is a chronic disease that reoccurs and delays healing, the main indicators of acute inflammatory response did not significantly improve in a short time. Therefore, more time is needed for observing the changes in serum indicators mentioned earlier.

### 4.2. The Effect of Moxibustion on TNF-*α*, IL-1*β*, *β*-EP, and Dyn in Serum of RA Patients

Our results showed that the levels of TNF-*α* and IL-1*β* in the treatment group decreased significantly after treatment, while there was no significant difference in the control group. The levels of *β*-EP and Dyn were significantly increased after treatment in the treatment group and superior to the control group. The correlation analysis results of the treatment group showed that the differences of *β*-EP and Dyn in the serum of patients were correlated with the differences of TNF-*α* and IL-1*β* after treatment, and the correlation was mainly negative.

Many cytokines active in the synovial membrane of RA patients are directly or indirectly involved in inflammatory pain. These cytokines play a crucial role in the pathogenesis of RA and can cause local inflammation, joint pain, and destruction [1]. TNF-*α* and IL-1*β*, as key inflammatory cytokines in the pathogenesis of RA, are highly expressed in the synovial fluid and serum of RA patients [19]. They can not only promote the formation of pannus and lead to the destruction of cartilage and bone but also produce other inflammatory factors such as IL-6 to aggravate joint inflammation that leads to the further increase of the pain. Hence, these two indicators can indirectly reflect the pain relief of RA. During the initial stage of pain signaling, TNF-*α* and IL-1*β* are released in large quantities. Opioid peptides can reduce the production of peripheral pain-causing substances such as cytokines (e.g., interleukins) and TNFs while providing peripheral analgesia [20, 21]. Therefore, the increase of peripheral opioid peptides may be closely related to TNF-*α* and IL-1*β*.

*β*-EP exerts analgesic effects on both the central and peripheral systems, with a significant effect on inflammatory pain. It can relieve pain by reducing the excitability of pain receptors, the transmission of action potential, and the release of proinflammatory neuropeptides from central and peripheral pain receptors of RA patients. *β*-EP is also involved in regulating inflammatory factors such as TNF-*α* and IL-1*β* by binding to immune cells' receptors [22]. Dyn plays an important role in pain regulation as a member of the endogenous opioid family and has a very complex mechanism. Dyn exerts an analgesic effect in both the central nervous system and the peripheral system. It can not only inhibit the release of cAMP, substance P, norepinephrine, and other neurotransmitters through the G-protein coupling mechanism to prevent the conduction of nerve impulses but also inhibit the activation of Na^+^ and K^+^ channels to prolong the action potential and achieve the analgesic effect.

Our study showed that moxibustion could enhance the role of conventional western medicine in improving the mobility of joints and reducing joint pain and swelling. Simultaneously, moxibustion can relieve pain by regulating the expression levels of TNF-*α*, IL-1*β*, *β*-EP, and Dyn in the serum of RA patients. This may be related to the effect of moxibustion on warming and dispersing cold, activating blood circulation and relieving pain. It can also be seen from the correlation analysis that *β*-EP and Dyn were correlated with TNF-*α* and IL-1*β* in the mechanism of action. With the increase of *β*-EP and Dyn, the contents of TNF-*α* and IL-1*β* decreased. Related studies confirmed that moxibustion could not only improve the RA expression levels of opioid peptides in the hypothalamus of rats and enhance the body's analgesia [23] but also significantly increase the pain threshold of rats with chronic visceral hyperalgesia by increasing the concentration of the Dyn in the spinal cord and inducing inhibition debris (including the postsynaptic inhibition and presynaptic inhibition), thereby blocking the further transmission of pain signals [24]. Meanwhile, moxibustion can stimulate immune cells to secrete more *β*-EP, increasing the contents of *β*-EP in the pituitary gland and peripheral lymph nodes of adjuvant arthritis rats [25]. These findings are consistent with the results of this study, which suggest that moxibustion therapy for RA can decrease TNF-*α* and IL-1*β* by increasing the levels of *β*-EP and Dyn in the body, thus playing an anti-inflammatory and analgesic role.

## 5. Conclusions

Moxibustion can improve the clinical symptoms of RA patients with conventional western medicine, which may be related to the effect of moxibustion on the levels of TNF-*α*, IL-1*β*, *β*-EP, and Dyn in the serum of RA patients. One of the effective mechanisms may be that moxibustion can affect the levels of *β*-EP and Dyn by downregulating the levels of inflammatory factors in the serum of RA patients, thus can control the degree of joint pain and swelling in these patients.

This study evaluated the effect of moxibustion on *β*-EP and Dyn as a pointcut to explore the anti-inflammatory and analgesic mechanism of moxibustion on RA patients. The correlation analysis of *β*-EP and Dyn with TNF-*α* and IL-1*β* showed a weak negative or a very weak positive correlation, which may be related to the small sample size and the slight difference in the degree of improvement in patients. Therefore, the sample size should be expanded in future relevant studies. In addition, this study indicated that moxibustion had anti-inflammatory and analgesic effects on RA. However, the mechanism of moxibustion's anti-inflammatory and analgesic effects on RA could be further studied because of its complexity.

## Figures and Tables

**Table 1:** Baseline characteristics.

Outcome measures	Treatment group (*n* = 30)	Control group (*n* = 31)	*P* value
Characteristic			
Gender, male/female	4/26	5/26	0.758^*∗*^
Age (y), mean (SD)	53.00 (8.80)	49.39 (7.72)	0.093^#^
Disease duration (y), mean (SD)	10.11 (9.02)	9.13 (9.07)	0.677^#^

Clinical symptoms			
VAS score, mean (SD)	6.47 (1.53)	6.10 (1.76)	0.38^#^
Morning stiffness score, mean (SD)	2.47 (2.01)	2.58 (1.80)	0.82^#^
DAS28 score, mean (SD)	5.33 (0.99)	5.50 (1.02)	0.49^#^

Routine inspection indexes			
RF (IU/ml), mean (SD)	209.60 (249.12)	190.95 (210.32)	0.75^▲^
ESR (mm/60 min), mean (SD)	65.40 (34.90)	64.61 (33.77)	0.93^▲^
CRP (mg/L), mean (SD)	20.21 (22.99)	18.01 (21.20)	0.70^▲^

^*∗*^ *P* value by *χ*^2^ test. ^#^*P* value by independent samples *t-*test. ^▲^*P* value by Mann–Whitney *U* test.

**Table 2:** Clinical symptoms and routine inspection indexes after treatment.

Outcome measures	Treatment group (*n* = 30)	Control group (*n* = 31)	*P* value
Clinical symptoms			
VAS score, mean (SD)	3.57 (1.49)	4.13 (1.77)	0.041^#^
Morning stiffness score, mean (SD)	1.27 (1.23)	2.06 (1.82)	0.045^#^
DAS28 score, mean (SD)	4.17 (0.94)	4.86 (1.40)	0.046^#^
Routine inspection indexes			

RF (IU/ml), mean (SD)	131.25 (156.01)	153.72 (178.35)	0.277^▲^
ESR (mm/60 min), mean (SD)	37.17 (27.42)	49.23 (28.55)	0.072^▲^
CRP (mg/L), mean (SD)	8.65 (9.48)	9.70 (14.00)	0.567^▲^

^#^ *P* value by independent samples *t-*test. ^▲^*P* value by Mann–Whitney *U* test.

**Table 3:** Changes of contents of TNF-*α*, IL-1*β*, *β*-EP, and Dyn.

Outcome measures	Treatment group (*n* = 30)	Control group (*n* = 31)	*P* value
TNF-*α* (pg/mL), mean (SD)			
Baseline	25.41 (12.01)	27.29 (14.45)	0.58
Posttherapy	20.04 (10.14)	25.45 (15.60)	0.045

IL-1*β* (pg/mL), mean (SD)			
Baseline	30.54 (13.97)	29.55 (14.66)	0.79
Posttherapy	24.39 (11.76)	28.26 (15.02)	0.031

*β*-EP (pg/mg), mean (SD)			
Baseline	11.42 (6.07)	12.84 (7.09)	0.41
Posttherapy	15.36 (10.56)	13.60 (8.68)	0.043

Dyn (pg/mg), mean (SD)			
Baseline	63.56 (35.85)	61.44 (25.20)	0.79
Posttherapy	78.30 (43.30)	65.54 (26.81)	0.042

*P* value by independent samples *t-*test.

**Table 4:** Correlation of *β*-EP and Dyn values with TNF-*α* and IL-1*β* values after treatment.

Outcome measures	Outcome measures	*r*	*P*
*β*-EP	TNF-*α*	**−0.119**	**0.531**
	IL-1*β*	**−0.361**	**0.050**
Dyn	TNF-*α*	**0.021**	**0.941**
	IL-1*β*	**−0.385**	**0.036**

## Data Availability

Data and materials from this trial are available upon reasonable request and approval by the corresponding author.
